# Supplementary material for: Antitumor Activity and Mechanism of Robustic Acid from Dalbergia benthami Prain via Computational Target Fishing
Source: Molecules. 2020 Aug 27;25(17):3919. doi: 10.3390/molecules25173919 (PMC7503938; doi:10.3390/molecules25173919)
Supplement: Supplementary file 1 [file molecules-25-03919-s001.pdf]

Supplementary material

Antitumor Activity and Mechanism of Robustic Acid from *Dalbergia benthami* Prain via Computational Target Fishing

Juanjuan Huang [1,2,†], Ying Liang [3,†], Wenyu Tian [3,†], Jing Ma [4], Ling Huang [3], Benjie Li[2], Rui Chen [1,2,*] and Dianpeng Li [1,5,*]

[1] School of Chemistry and Chemical Engineering, Guangxi University, Nanning 530004, China; hjjcbw@163.com

[2] Faculty of Chinese Medicine Science, Guangxi University of Chinese Medicine, Nanning 530222, China; lbjjie15@163.com

[3] School of basic Medical Sciences, Guangxi Medical University, Nanning 530021, China; liangtty@163.com (Y.L.); bcyy15777199540@163.com (W.T.); huangling0916@hotmail.com (L.H.)

[4] School of basic Medical Sciences, Guangxi University of Chinese Medicine, Nanning 530200, China; weixin0815@163.com

[5] Guangxi Key Laboratory of Functional Phytochemicals Research and Utilization, Guangxi Institute of Botany, Guangxi Zhuang Autonomous Region and Chinese Academy of Sciences, Guilin 541006, China.

* Correspondence: 58251323@163.com (R.C.); ldp@gxib.cn or phytoldp@hotmail.com (D.L.); Tel.: +86-0771-4735732 (R.C.); +86-0773-3550682 (D.L.)

† These authors contributed equally to this work.

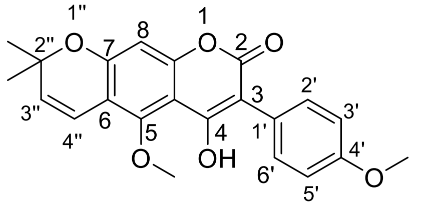

1

Robustic acid: white solid, productivity: 40%; m.p. 208-20 °C; HR-MS(ESI) *m/z*: calculated for $C_{22}H_{20}O_6$ $[M + Na]^+$: 403.1157, found: 403.1157. ^{1}H-NMR (400 MHz, CDCl3) δ 9.97 (s, 1H, 4-OH), 7.46 (dd, 2H, *J* =11.4 Hz, 2.9 Hz, H-2', H-6'), 6.97 (dd, 2H, *J* =8.7 Hz, 2.9 Hz, H-3', H-5'), 6.63 (s, 1H, H-8), 6.50 (d, 1H, *J* =10.0 Hz, H-4''), 5.77 (d, 1H, *J* =10.0 Hz, H-3''), 3.96 (s, 3H, 4'-OCH3), 3.77 (s, 3H, 5-OCH3), 1.48 (s, 6H, -CH3); ^{13}C-NMR (125 MHz, CDCl3) δ 162.07 (C-4), 160.96 (C-2), 158.83 (C-4'), 157.97 (C-7), 153.65 (C-5), 152.11 (C-9), 131.78 (C-4''), 131.43 (C-2', 6'), 124.92 (C-6), 123.21 (C-1'), 115.03 (C-3''), 113.71 (C-3', 5'), 110.67 (C-8), 103.83 (C-10), 101.50 (C-3), 78.33 (C-2''), 65.19 (3'-OCH_3), 55.42 (5-OCH_3), 27.96 (2-2×CH_3).
